# Glut1 deficiency syndrome throughout life: clinical phenotypes, intelligence, life achievements and quality of life in familial cases

**DOI:** 10.1186/s13023-022-02513-4

**Published:** 2022-09-24

**Authors:** Sara Olivotto, Alessandra Duse, Stefania Maria Bova, Valeria Leonardi, Elia Biganzoli, Alberto Milanese, Cristina Cereda, Simona Bertoli, Roberto Previtali, Pierangelo Veggiotti

**Affiliations:** 1 Pediatric Neurology Unit, Vittore Buzzi Children’s Hospital, Via Castelvetro, 32, 20154 Milan, Italy; 2 University of Milan, Via Festa del Perdono 7, 20122 Milan, Italy; 3 Department of Biomedical and Clinical Sciences, L. Sacco, University of Milan, Via Giovanni Battista Grassi, 74, 20157 Milan, Italy; 4 Medical Statistics Unit, Department of Biomedical and Clinical Sciences L. Sacco, “Luigi Sacco” University Hospital, Università degli Studi di Milano, Milan, Italy; 5 Laboratory of Medical Genetic, “Vittore Buzzi” Children’s Hospital, Via Castelvetro, 32, 20154 Milan, Italy; 6 Obesity Unit-Laboratory of Nutrition and Obesity Research, Department of Endocrine and Metabolic Diseases, IRCCS Istituto Auxologico Italiano, Via Ariosto 13, 20145 Milan, Italy; 7 International Center for the Assessment of Nutritional Status (ICANS), Department of Food, Environmental and Nutritional Sciences (DeFENS), University of Milan, 20133 Milan, Italy

**Keywords:** Glut1 deficiency syndrome, Familial GLUT1-DS, Quality of life, Life achievement, IQ

## Abstract

**Background:**

Glut1 deficiency syndrome (Glut1-DS) is a rare metabolic encephalopathy. Familial forms are poorly investigated, and no previous studies have explored aspects of Glut1-DS over the course of life: clinical pictures, intelligence, life achievements, and quality of life in adulthood.

Clinical, biochemical and genetic data in a cohort of familial Glut1-DS cases were collected from medical records. Intelligence was assessed using Raven’s Standard Progressive Matrices and Raven’s Colored Progressive Matrices in adults and children, respectively. An ad hoc interview focusing on life achievements and the World Health Organization Quality of Life Questionnaire were administered to adult subjects.

**Results:**

The clinical picture in adults was characterized by paroxysmal exercise-induced dyskinesia (PED) (80%), fatigue (60%), low intelligence (60%), epilepsy (50%), and migraine (50%). However, 20% of the adults had higher-than-average intelligence. Quality of Life (QoL) seemed unrelated to the presence of PED or fatigue in adulthood. An association of potential clinical relevance, albeit not statistically significant, was found between intelligence and QoL. The phenotype of familial Glut1-DS in children was characterized by epilepsy (83.3%), intellectual disability (50%), and PED (33%).

**Conclusion:**

The phenotype of familial Glut1-DS shows age-related differences: epilepsy predominates in childhood; PED and fatigue, followed by epilepsy and migraine, characterize the condition in adulthood. Some adults with familial Glut1-DS may lead regular and fulfilling lives, enjoying the same QoL as unaffected individuals. The disorder tends to worsen from generation to generation, with new and more severe symptoms arising within the same family. Epigenetic studies might be useful to assess the phenotypic variability in Glut1-DS.

**Supplementary Information:**

The online version contains supplementary material available at 10.1186/s13023-022-02513-4.

## Background

Glut1 deficiency syndrome (Glut1-DS) is a rare metabolic encephalopathy associated with abnormal brain metabolism. Impaired glucose transport at the level of the endothelial cells of the blood brain barrier [1, 2] leads to decreased glucose supply to the brain, which in turn results in brain functional alteration [3]. Genetically, the disease is due to pathogenic variant in the *SLC2A1* gene that can be sporadic or inherited in autosomal dominant fashion, but rare cases of autosomal recessive transmission have been reported [4].

Clinical picture is characterized by seizures appearing early in life and often drug resistant, movement disorders (MDs) and intellectual disability [5]. According to clinical manifestations, patients with Glut1-DS can be classified into four phenotypic groups: minimal, mild, moderate and severe [6]. Clinical presentations in familial cases are heterogeneous and usually less severe [7], mostly due to missense pathogenic variant.

There are few reports dealing with the signs and symptoms of Glut1-DS in adulthood, and little is known about the disease course [8]. The clinical phenotype differs according to age [9], a still poorly understood phenomenon that probably involves a change in the rate of glucose utilization in the brain. This rate increases from birth until the age of about 4 years, remains high until the age of 10, and thereafter declines, reaching the adult level by the age of 16–18 [10]. This pattern recalls the evolution of epilepsy: in the classical phenotype, seizures start in the first months of life [5, 8] and, if not treated correctly, become progressively more severe until the end of childhood. In adolescence, seizure frequency declines and, in some cases, spontaneous recovery can occur [5, 8], irrespective of treatment [8]. In patients in whom epileptic events decline or even disappear during adolescence, paroxysmal MDs, especially paroxysmal exercise-induced dyskinesia (PED), either appear or, if already present, worsen [8, 9, 11]. Cognitive functions remain stable throughout life [8].

With the aim of addressing a gap in the literature, we set out to describe clinical phenotypes, intelligence, life achievements (in terms of education, work, social relationships, and autonomy), and quality of life (QoL) in a sample of families with genetically confirmed familial Glut1-DS diagnosed at the Pediatric Neurology Unit at “Vittore Buzzi” Children’s Hospital, Milan, Italy.

## Results

**Fig. 1:**
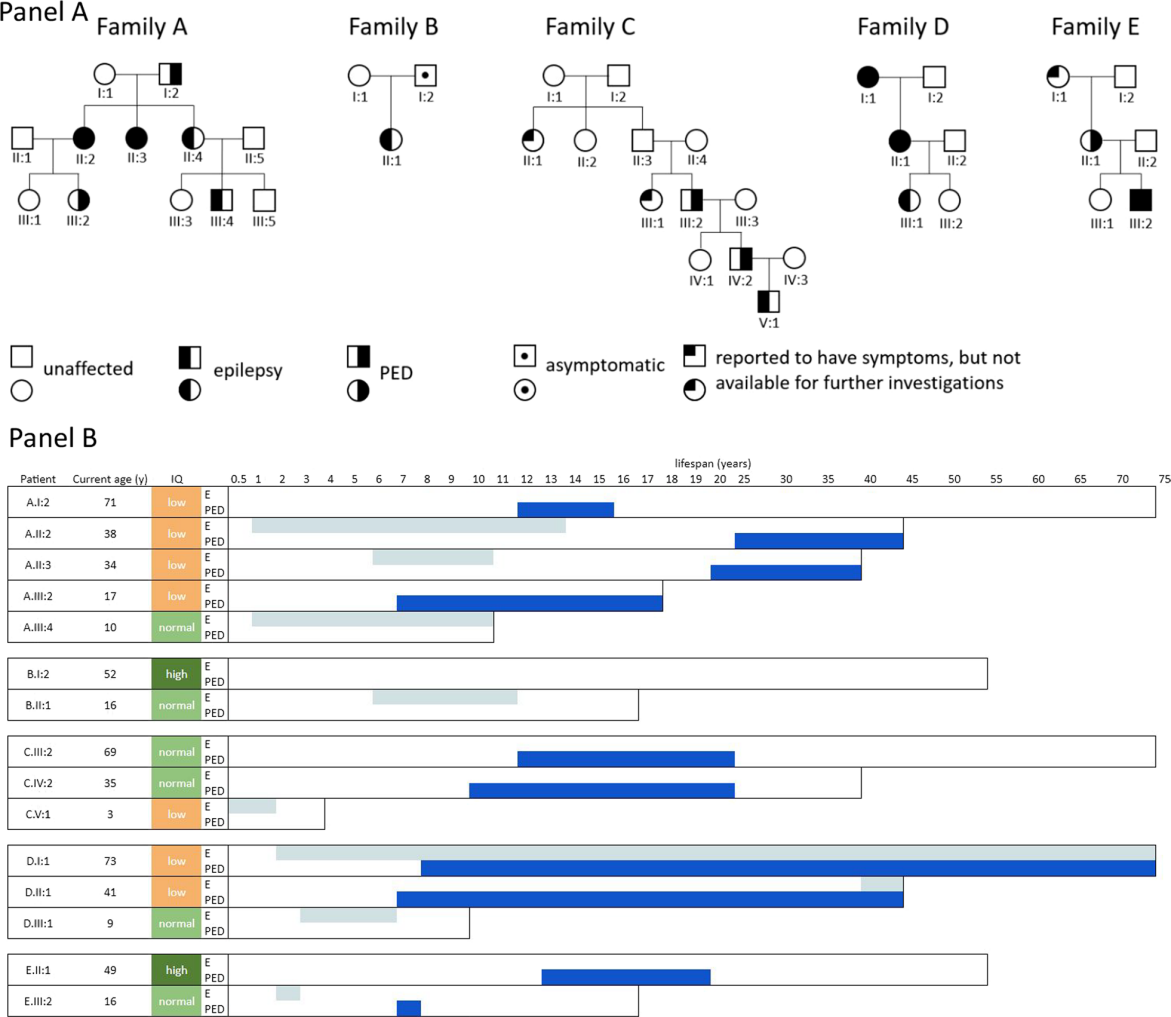
Family trees and clinical manifestation. Panel** A**: Family trees of the five families. Panel** B**: Duration of epilepsy (E) and of paroxysmal exertion-induced dyskinesia (PED) throughout lifespan is shown by gray and blue lines respectively. For each patient, current age and intelligence quotient are reported. Abbreviations: *y* years

We identified 16 subjects with familial Glut1-DS. They came from five families in which the disease was diagnosed in up to three generations; 15 of the 16 subjects were available for the second part of the study. The family trees are reported in Fig. 1- panel A.

### Clinical phenotype

The subjects’ demographic, genetic and clinical data, and neurological signs and symptoms are detailed in Tables 1, 2.

**Table 1:** Clinical manifestations

Patients	A.I:2	A.II:2	A.II:3	A.II:4	A.III.2	A.III.4	B.I:2	B.II:1	C.III:2	C.IV:2	C.V:1	D.I:1	D.II:1	D.III:1	E.II:1	E.III:2
Sex	M	F	F	F	F	M	M	F	M	M	M	F	F	F	F	M
Current age (years)	71	38	34	31	17	10	52	16	69	35	3	73	41	9	49	16
Genetic pathogenic variant																
Coding	c.493G > A	c.493G > A	c.493G > A	c.493G > A	c.493G > A	c.493G > A	c.26C > T	c.26C > T	c.1363A > G	c.1363A > G	c.1363A > G	c.388G > A	c.388G > A	c.388G > A	c.200 T > C	c.200 T > C
Protein	p.Val165Ile	p.Val165Ile	p.Val165Ile	p.Val165Ile	p.Val165Ile	p.Val165Ile	p.Thr9Met	p.Thr9Met	p.Thr455Ala	p.Thr455Ala	p.Thr455Ala	p.Gly130Ser	p.Gly130Ser	p.Gly130Ser	p.Leu67Pro	p.Leu67Pro
ACMG criteria	Pathogenic	Pathogenic	Pathogenic	Pathogenic	Pathogenic	Pathogenic	VUS/Likely pathogenic	VUS/Likely pathogenic	Likely pathogenic	Likely pathogenic	Likely pathogenic	Pathogenic	Pathogenic	Pathogenic	VUS/Likely pathogenic	VUS/Likely pathogenic
CSF/blood glucose ratio	NA	0.44	NA	NA	0.38	0.42	NA	0.52	NA	NA	0.37	NA	NA	0.39	NA	0.5
Epilepsy																
Age at onset (years)	(no epilepsy)	1	6	6	(no epilepsy)	1	(no epilepsy)	6	(no epilepsy)	(no epilepsy)	0.5	17	2	3	(no epilepsy)	2
Seizure frequency	–	Every 2–3 days	Every 2–3 weeks	Every 2–3 months	–	Every 2 months	–	Every day	–	–	Every day	Every 2–3 months	Every month	Every month	–	Every year
Seizures	–	Abs, GTC (stopped at 13y)	Abs (stopped at 10y)	GTC (stopped 20y)	–	GTC, Abs	–	Abs (stopped at 11y with KD)	–	–	FS (stopped at 1y with KD)	GTC	GTC (stopped at 20y relapsed at 39y)	GTC (stopped at 6y with KD)	–	Abs (stopped at 2y)
Movement disorders																
PED	Yes	Yes	Yes	No	Yes	No	No	No	Yes	Yes	No	Yes	Yes	No	Yes	Yes
Limbs affected	Lower limbs	Lower limbs	Lower and upper limbs	–	Lower limbs	–	–	–	Lower limbs	Lower and upper limbs	–	Lower limbs	Lower and upper limbs		Lower limbs	Lower limbs
Age at onset (years)	12	25	20	–	7	–	–	–	12	10	–	8	7	–	13	7
Maximum Frequency	every month	Every 2–3 weeks	Every 2–3 weeks	–	Every day	–	–	–	Every month	Every day	–	NA	Every month	–	Every month	Every week
Progression of PED over time	improvement after 15y	Improvement	Worsening	–	Worsening	–	–	–	Improvement after 20y	Improvement after 20y	–	NA	Improvement	–	Improvement after 20y	Improvement after KD
Remission	Yes	No	No	–	No	–	–	–	Yes	No	–	No	Yes	–	Yes	Yes
Abnormal eye movements	No	No	No	No	No	No	No	No	No	No	Yes	No	No	No	No	No
Other symptoms																
Migraine	No	Yes	Yes	No	No	No	No	Yes	Yes	No	No	Yes	No	No	Yes	No
Fatigue	No	Yes	Yes	No	No	No	No	Yes	Yes	Yes	N	Yes	Yes	No	No	No
Acute transient focal neurological disturbance	No	Yes	Yes	No	No	No	No	No	No	No	No	No	Yes	No	No	No
Psychiatric disorders	No	Depression	Panic attack	No	No	ADHD	No	No	No	No	No	No	No	No	No	No
Ongoing therapy																
ASMs	No	Yes	No	Yes	No	Yes	No	No	No	No	No	Yes	Yes	No	No	No
KD	No	No	No	No	Yes (start 6y stop at 10y)	No	No	Yes (start 9y)	No	No	Yes (start 1y)	No	No	Yes (start 6y)	No	Yes (start 10y)

*M* male, *F* female, *CSF* cerebrospinal fluid, *NA* not available, *y* years, *KD* ketogenic diet, *Abs* absences, *GTC* generalized tonic–clonic seizures, *FS* focal seizures, *PED* paroxysmal exertion-induced dyskinesia, *ADHD* attention deficit hyperactivity disorder, *ASMs* anti-seizure medications

**Table 2:** Intelligence level, Life achievements and quality of life

Patients	A.I:2	A.II:2	A.II:3	A.II:4	A.III.2	A.III.4	B.I:2	B.II:1	C.III:2	C.IV:2	C.V:1	D.I:1	D.II:1	D.III:1	E.II:1	E.III:2
Intelligence level																
Percentile on Raven’s test	15th	13th	17th	16th	5th	25th	99th	50th	33rd	45th	2nd	14th	24th	15th	93rd	51st
Life achievements																
Education level	Illiterate	Middle school	Middle school	NA	High school	Primary school	High school	High school	High school	High school	Kindergarten	Middle school	Middle school	Primary school	Degree	High school
Special needs teacher	/	No	No	NA	Yes	Yes	No	No	No	No	Yes	No	No	Yes	No	Yes
Learning difficulties	NA	Yes	Yes	NA	Yes	Yes	No	No	No	No	NA	Yes	Yes	No	No	Yes
Rehabilitation	No	No	No	NA	No	PSM	No	No	No	No	PSM	No	No	PSM + ST	No	PSM + ST
Social Life																
Married	Yes	Yes, divorced	Yes, divorced	NA	NA	NA	Yes	NA	Yes	Yes	NA	Yes	Yes, divorced	NA	Yes	NA
Job	Farmer, bricklayer. Now retired	Unemployed	Caregiver	NA	NA	NA	Computer scientist	NA	Chemical expert, now retired	Chemical expert	NA	Unemployed (never had job)	Janitor	NA	Social service	NA
Part time / full time/ occasional	full time and part time	/	Occasional	NA	NA	NA	Full time	NA	Full time	Full time	NA	/	Part- time	NA	Full time	NA
Driving license	No	Yes	No	NA	NA	NA	Yes	NA	Yes	Yes	NA	No	Yes	NA	Yes	NA
QoL domains																
Physical	41.67	87.5	10.41	NA	–	–	95.83	–	52.083	83.33	–	60.42	70.83	–	75	–
Psychological	60	66.25	56.25	NA	–	–	93.75	–	60	70	–	62.5	67.5	–	78.75	–
Independence	45.31	90.62	68.75	NA	–	–	93.75	–	60.94	82.81	–	59.37	60.94	–	92.19	–
Social relationships	54.17	83.33	77.08	NA	–	–	95.83	–	58.33	83.33	–	54.17	56.25	–	83.33	–
Environmental	53.13	68.75	55.47	NA	–	–	92.97	–	64.06	72.66	–	64.07	65.62	–	78.12	–
Spirituality	31.25	75	81.25	NA	–	–	100	–	50	75	–	93.75	75	–	75	–

Data on intelligence level and life achievements are available for 9 adults and 6 children. Quality of life domains are shown for 9 adults, with scores converted into a scale of 0–100. (Abbreviations: NA, not available; PSM, psychomotor therapy; ST, speech therapy)

Ten adults (60% females) aged from 31 to 73 years (median age: 45 years) and six children (50% females) aged from 3 to 17 years (median age: 13 years) were identified and included. Glut1-DS diagnosis was genetically confirmed. Cerebrospinal fluid (CSF)/blood glucose ratio was calculated in 1/10 adults (10%) and in 100% of the children. All those analyzed had a CSF/blood glucose ratio lower than 0.60 (mg/dl).

Epilepsy was reported in 5/10 (50%) adults. The first seizure occurred at between 1 and 17 years of age (median: 6 years). Nine adults (90%) are currently seizure free. Epilepsy was documented in 5/6 (83.3%) children, with an age at onset ranging from 6 months to 6 years (median: 2 years). Four children (67%) are currently seizure free.

Paroxysmal exercise-induced dyskinesia was reported in 8/10 (80%) adults with an age at onset of 7–25 years (median: 12 years). In 5/8 (62.5%) cases, PED was found to involve only the lower limbs, whereas in 3/8 (37.5%) it affected both the upper and the lower limbs. The maximum reported frequency of PED was daily in 1/8 (12.5%), every 2–3 weeks in 2/8 (25%), and every month in 4/8 (50%) patients; in one patient this information was not available. In 6/8 (75%) patients, PED improved over time, showing decreased frequency and intensity; in 1/8 (12.5%) it worsened, and in the other the relevant information was not available. Four adults (50%) achieved complete remission of PED.

Two (33%) children had PED affecting the lower limbs, in both cases with onset at the age of 7 years. At its maximum frequency, PED occurred daily in one child and weekly in the other; one patient worsened during adolescence, whereas the other, after starting a ketogenic diet (KD), improved, eventually reaching remission. The clinical course of epilepsy and PED over time is shown in Fig. 1—panel B. Ataxia was reported in 1/10 (10%) adults. Abnormal eye movements were absent in the adults and reported in 1/6 (16.7%) of the children.

Migraine was present in 5/10 (50%) adults and one child (16.7%). Fatigue was reported in 6/10 (60%) adults and one child (16.7%). Three adults (30%) experienced acute onset of focal neurological disturbances followed by complete remission within 24 h; none of the children experienced such episodes.

Neuropsychiatric disorder**s** were reported in 2/10 adults (20%): depression in one and panic attacks in the other; one child (16.7%) had a diagnosis of attention deficit hyperactivity disorder (ADHD).

Overall, one adult (10%) was symptom free, whereas none of the children was completely asymptomatic**.**

As regards therapy, 4/5 adults with epilepsy (80%) are currently on anti-seizure medications (ASMs); none has ever tried a KD; 3/5 (60%) are currently seizure free, including the patient under pharmacological treatment for epilepsy. One child with epilepsy (20%) is on ASMs and 4/5 (80%) are on a KD.

### Intelligence, life achievements and QoL

Tables 1 and 2 presents the results of the intelligence testing, the investigation of life achievements, and the QoL questionnaires.

Intelligence was assessed using Raven’s Standard Progressive Matrices (SPM) in adults and Raven’s Colored Progressive Matrices (CPM) in children. Intelligence in the adults ranged from the 10th percentile to the 99th percentile (median 20.5th percentile). Six (60%) were found to have low, 2/10 (20%) average, and 2/10 (20%) high intelligence.

Among the children, intelligence percentiles ranged from the 2nd to the 51st (median: 20th); 3/6 (50%) showed low and three (50%) average intelligence.

Life achievements were assessed through an ad hoc interview (Additional file 1). Data were available for 9/10 adults. As regards education, 1/9 (11.1%) received no schooling at all; 4/9 (44.4%) finished middle school; 3/9 (33.3%) completed high school; and 1/9 (11.1%) had a degree. Among those who attended school, 4/8 (50%) reported difficulties but none was ever assisted by a special needs teacher or received rehabilitation. All married, and 3/9 (33.3%) are now divorced. With regard to work, 8/9 (88.8%) reported having worked during their lives: 2/8 (25%) are now retired; one (12.5%) unemployed; one (12.5%) has a part-time job; one (12.5%) has occasional jobs; and 3/8 (37.5%) work full time. Six of the nine patients (66.7%) have a driving license.

All the children surveyed currently attend school; 5/6 (83.3%) have special needs teachers; and 4/6 (66.7%) are receiving or have received some form of rehabilitation, such as speech or psychomotor therapy. Learning difficulties, evaluated in the children aged over 6 years, were reported in 3/5 (60%).

Quality of life was assessed, using the *World Health Organization Quality of Life Questionnaire* (WHOQOL-100, Italian version), in nine adults. *Physical* domain values ranged from 10.41 to 95.83 (median = 70.83; IQR = 31.25); *Psychological* domain values from 56.25 to 93.75 (median = 66.25; IQR = 10); *Independence* domain values from 45.31 to 93.75 (median = 68.75; IQR = 29.68); *Social Relationship* domain scores from 54.17 to 95.83 (median = 77.08; IQR = 27.08); *Environmental* domain values from 53.13 to 92.97 (median = 65.62; IQR = 8.60); and *Spirituality* domain scores from 31.25 to 100 (median = 75.00; IQR = 6.25).

### QoL in adults with Glut1-DS: correlation with clinical parameters

Principal component analysis (PCA) of the data recorded in the subgroup of adults (n = 9) revealed that all six QoL domains were positively correlated with each other and with dimension 1 of the PCA (i.e., the x-axis of the plot). This can be observed in Additional file 2, where the arrows representing these domains show a tendency to group towards the right side of the graph, with dimension 1 of the PCA map accounting for 73.93% of the total explained variability.

The correlation between the domains made it desirable to reduce the number of the outcome variables from the initial six (six domains) to a single variable capable of synthesizing all the information (identified as the arithmetic mean of the aforementioned six variables). Correlations across the six QoL domains were found to be strong on average, with Pearson’s correlation coefficients ranging from 0.38 to 0.96; for further details of the PCA analysis, see Additional file 3.

​​The next step was to evaluate the relationship between the single outcome variable, defined as the synthesis of the six QoL domains (QoL-S), and the following variables: intelligence quotient (IQ) (low, average, high), PED (yes / no), fatigue (yes / no).

**Fig. 2:**
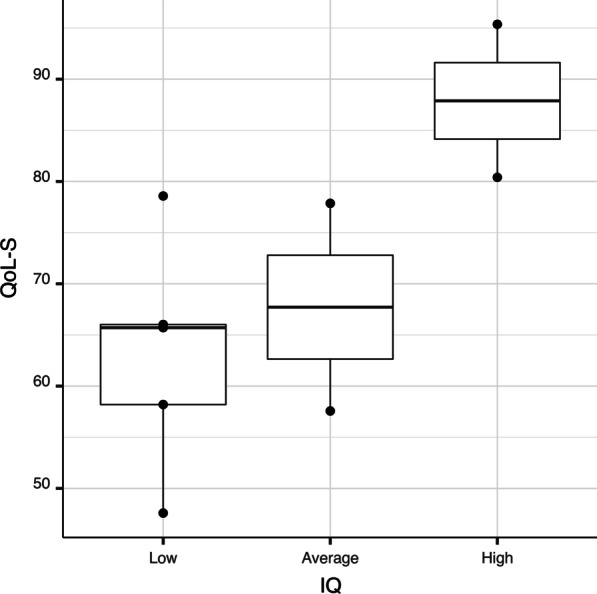
Boxplot of IQ and QoL. A potentially clinically relevant difference in the QoL variables seems to emerge between the patients with "high" versus those with "low"/"average" IQ

Although the association between QoL-S and IQ was not statistically significant (*p* = 0.123), a potentially clinically relevant difference in the QoL-S variables was found between the patients with high IQ (median = 87.88) and those with low to average intelligence (median 65.71 to 67.72, respectively), as shown in Fig. 2.

Statistical tests to detect associations of both PED and Fatigue with QoL-S also yielded non-significant results (*p* = 0.121 and *p* = 0.439, respectively). (Additional files 4, 5).

### QoL in adults with Glut1-DS: comparison with a normal population

The adults affected by Glut1-DS were compared, for QoL, with a group of healthy subjects, matched for age and gender with the patients (Table 3).

**Table 3:** Descriptive analysis of cases (n = 9) and age-sex matched controls (n = 18)

Variables	Cases (n = 9)	Controls (n = 18)
Sex = Male	4 (44.4)	8 (44.4)
Age	49.00 [38.00, 69.00]	49.00 [38.00, 69.00]
PED = Yes	4 (44.4)	0 (0.0)
Fatigue = Yes	6 (66.7)	0 (0.0)
Physical domain	70.83 [52.08, 83.33]	76.04 [60.94, 81.25]
Psychological domain	66.25 [60.00, 70.00]	72.50 [64.69, 77.81]
Independence domain	68.75 [60.94, 90.62]	81.25 [70.31, 85.94]
Social relationships domain	77.08 [56.25, 83.33]	69.79 [65.10, 75.00]
Environmental domain	65.62 [64.06, 72.66]	72.66 [68.16, 78.71]
Spiritual domain	75.00 [75.00, 81.25]	75.00 [64.06, 84.38]

The evaluation of putative differences in QoL scores between cases and controls was performed through conditional logistic regression models, using the individual’s status (case/control) as the response variable and the single QoL domain scores as explanatory variables. Table 4 shows the results of the univariate conditional logistic models, reported as odds ratios (ORs) and 95% confidence intervals; no variable showed statistical significance.

**Table 4:** Univariate conditional logistic regression models

QoL Domain variable	OR EST (95% C.I.)	*p* value
Physical	0.973 (0.926–1.023)	0.285
Psychological	0.995 (0.929–1.067)	0.894
Independence	0.947 (0.870–1.030)	0.202
Social relationships	1.013 (0.951–1.078)	0.696
Environmental	0.944 (0.857–1.040)	0.243
Spiritual	0.994 (0.950–1.039)	0.777

Univariate conditional logistic regression performed using the 6 QoL domains as explanatory variables and case/control status as outcome variable

In order to identify possible patterns of QoL in the two groups, a PCA of the data was performed. The first two dimensions were found to account for 74.61% (the first axis 58.34% and the second 16.27%) of the total variability of the dataset.

**Fig. 3:**
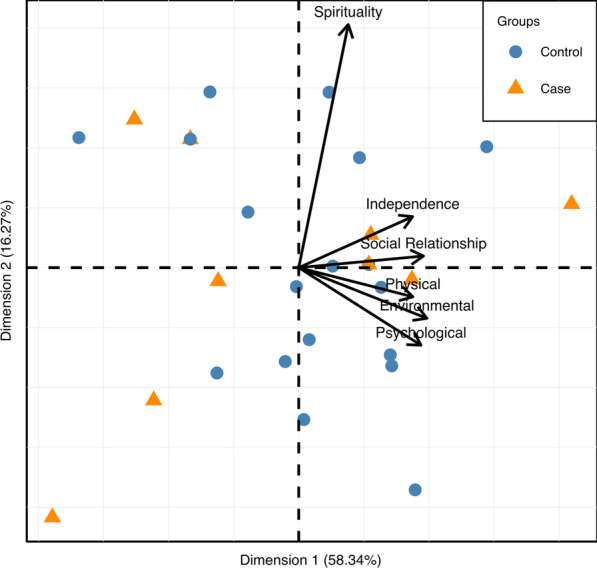
PCA Biplot graph. Vectors represent the active variables (QoL domains) while dots represent individuals: orange triangles represent Glut1-DS patients (cases) and blue dots represent age- and sex-matched healthy subjects (controls)

On the biplot graph (Fig. 3), all the variables considered, with the exception of “Spiritual domain”, were found to be correlated with each other and with dimension 1 of the PCA (i.e., the x-axis of the plot). The variable “Spiritual domain” was uncorrelated with the previous ones and correlated with dimension 2, bringing additional and different information compared to that conveyed by the other variables considered.

In addition to the relationship between the variables, the biplot graph also considered individual subject data: in particular, the individuals were classified according to their status as cases/controls (shown by a color code in the plot). However, no obvious clustering of the subjects emerged.

## Discussion

The present study of familial Glut1-DS focused on five families with up to three affected generations. We analyzed the clinical picture in children and adults and reported the evolution of the disease over time and how it has affected adult patients’ QoL and life achievements. The literature to date offers little information about the natural evolution of Glut1-DS beyond childhood and in relatives of different ages [7, 8, 11]. We found great clinical variability between patients, including individuals belonging to the same family. We did not find specific correlation between clinical severity and CSF/blood ratio, moreover the small number of patients tested with lumbar punction, do not allow to perform statistical studies.

Epilepsy occurred in 62.5% of our cohort, with a median age at onset of 2 years. Seizures were reported in 50% of the adult cases (median age at onset: 6 years). Epilepsy was found to be more frequent in the children than the adults, with onset tending to occur earlier than in their parents/grandparents.

Movement disorders were the most prominent clinical feature in the adult patients, consisting almost exclusively of PED (88% of affected individuals). PED was observed in one third of the children. The median age at onset of PED, like epilepsy, was found to be lower in the children (7 years) than in the adults (12 years). However, we had only two pediatric cases of PED, and a cohort this small obviously limits possible statistical speculation. In most cases, PED, being a paroxysmal disorder induced by movement, interferes little with autonomy in daily life and tends to improve spontaneously over time. One subject presented ataxia.

In other regards, too, our results are in line with the literature [7], according to which the first symptom recorded is usually epilepsy, which is the most prominent feature during childhood, often being the major symptom leading to the Glut1-DS diagnosis. In many cases, seizures tend to decrease or cease, either after the introduction of a KD or, later on, spontaneously. The rate of epilepsy is lower among adults, even though they rarely follow a KD [7, 8, 11]. MDs typically appear or worsen in early adolescence, when they may constitute the most disabling feature, even when not associated with other disorders. In early adulthood, movement disorders often improve in terms of frequency even though it does not necessarily disappear over time and characteristically worsens in stressful or physically demanding situations [7, 11].

Fatigue and migraine are known to be associated with Glut1-DS [7, 8, 11]. In our cohort, fatigue was reported by 60% and migraine by 50% of adult patients. Therefore, together with PED and epilepsy, they are the most frequent associated disorders in adults with Glut1-DS. Fatigue and migraine were, instead, rarely reported in the children (16.7% and 17% respectively).

Three adults (30%) presented an acute transient focal neurological disturbance with spontaneous regression. This neurological condition is a newly identified feature of Glut1-DS that needs to be carefully investigated and characterized. Its identification opens the possibility of including Glut1-DS among the causes of transient acute neurological deficits in adults, and thus of increasing diagnostic accuracy in this setting, even in patients with poorly characterized or no other symptoms.

Psychiatric disorders, a known comorbidity of Glut1-DS [9, 12], seemed to be age dependent in our study, being found to consist of depression and panic attacks in adults and ADHD in children.

In line with previous research by our group [13], intelligence was frequently below normal range across our entire cohort: only four subjects (two adults and two adolescents) showed average intelligence, while just two showed higher-than-average intelligence. These subjects belonged to three families. This is the first report of high intellectual function in Glut1-DS patients. One of the two with high IQ never displayed any symptoms; the other showed lower limb PED from adolescence until the age of 20 years. It should be emphasized that none of the aforementioned subjects has ever tried a KD. Overall, the children in this study showed lower intelligence than their parents and grandparents, suggesting a generation-by-generation decrease in cognitive functioning.

The data on life achievement in adulthood showed that all the patients got married, indicating that social functioning was such that a satisfactory relationship could be established and continued. Two thirds (66.7%) of adults had a driving license, implying the ability to pass a standard driving test and therefore enjoy total autonomy in traveling.

Taken as a whole, these observations show at least some familial Glut1-DS adults can enjoy regular and fulfilling lives. This agrees with the findings of Klepper et al. [9], who showed that social adaptive behavior in adult patients is often well developed, apparently allowing patients to lead normal daily lives.

QoL has never previously been analyzed in adults with Glut1-DS. Our investigation shows that this population has a similar QoL to the general population, as documented by the absence of statistical differences in QoL between patients and a sex- and age-matched control group.

Moreover, no relationship was found between adult patients’ QoL and the two most frequent neurological symptoms: PEDs and fatigue. A potentially clinically relevant difference in the QoL variables, albeit not statistically significant (*p* = 0.123), emerged between the group with high intelligence and the patients with low/normal intelligence. This finding, suggesting that high intelligence can act as a protective factor in terms of QoL, emphasizes the importance of early implementation tools geared at promoting better QoL, also in asymptomatic or paucisymptomatic subjects.

Albeit in a small sample, the present data on Glut1-DS in families with affected individuals of different ages suggest that a clinical deterioration (of epilepsy, MDs and intelligence) may occur from generation to generation. This hypothesis, while based on clinical experience, seems to be supported by a series of considerations.

First, all the adult patients were diagnosed with Glut1-DS after their children and/or grandchildren had received the same diagnosis, and none of them had ever previously undergone medical investigations prompted by neurological symptoms. In other words, while Glut1-DS had never been suspected in the adults, their children or grandchildren came to medical attention very early. This clinical observation supports the idea that Glut1-DS symptoms can arise increasingly early in successive generations. However, it would be necessary to evaluate further possible generations within these family groups; therefore, if possible, follow-up of current patients and possible subsequent generations will be maintained. Moreover, we should consider a potential inclusion bias in adults, since a population of adults with mild symptoms may be underdiagnosed if they have mildly affected children, who will not come to medical attention.

Second, even though similar proportions of adults and children had or still have educational and developmental difficulties, no adult ever had a special needs teacher or received rehabilitation. Conversely, 83.3% of the children receive some help at school and 66.7% are involved in rehabilitation programs. These data should be interpreted in the light of the different periods in which the two groups grew up, given that they are characterized by different approaches to neuropsychiatric disorders and support strategies. However, this also means that these adult patients’ academic and work achievements were reached without any support. On the other hand, the sum of cognitive difficulties, clinical symptoms, and sometimes psychological fragility can, in the absence of treatment or adequate support, exacerbate social issues and eventually lead to impaired personal relationships, job insecurity, and poor management of clinical aspects of the disease. This consideration reinforces the importance of a correct diagnosis in asymptomatic/paucisymptomatic adults.

Third, mild and moderate forms of the disease are most likely associated with missense *SLC2A1* variants resulting in 50–70% residual function of GLUT1 transporter. However, genotype–phenotype correlation remained elusive with high interindividual phenotypic variability, even between mutated members of the same family [14]. Moreover, 2 out of 5 identified *SLC2A1* mutations (40%) are located on transmembrane helix 4 (TMH4) and transmembrane helix 5 (TMH5) encoded by exon 4, confirming a mutational hotspot in the *SLC2A1* gene. [15]. All the patients in our families have missense mutations and then they should belong to minimal and mild phenotypic groups [6]. In particular, the patients in family A and family D with a mutation within exon 4, considered a mutational hot spot, present a more severe phenotype [15].

More strictly genomic consideration of the condition and further molecular studies are needed to explain the progression of the severity through the generations. Epigenetic studies might be useful to assess the phenotypic variability in Glut1-DS. For example, DNA methylation could be implicated in progressive lowering of the age at epilepsy onset [16, 17].

Improving our knowledge of this syndrome will help to clarify the genetic mechanisms underlying a possible generational deterioration. It remains to be established whether adult patients should be treated with a KD, and whether this intervention can help to improve the evolution of the disease. Increased knowledge of Glut1 disease, especially in adulthood, could allow us to diagnose paucisymptomatic cases and provide families with the right genetic advice.

### Study limitation

This study presents some limitations. First, Glut1-DS is a rare disorder and therefore the study sample is small. Furthermore, previous research studies are scarce or inconsistent, so we had little literature with which to compare our research.

Finally, adult patients were examined by expert physicians only when their children/grandchildren came to medical attention; therefore their medical history could be collected, from the patients themselves, only retrospectively, and the nature of their clinical conditions during childhood could not be established with precision.

## Conclusions

Two aspects must be considered in the evaluation and counseling of a patient affected by familial Glut1-DS. The first is that each symptom has its own typical age at onset and trend: epilepsy predominates in childhood, while MDs may arise in adolescence. PEDs and fatigue, followed by epilepsy and migraine, are the most frequent manifestations in adulthood. Over time and in old age the symptoms often abate, and at least a subset of familial Glut1-DS adults can lead regular and fulfilling lives. However, especially in the presence of cognitive difficulties or poorly managed clinical disorders, difficulties in the work and social spheres may arise and compromise the patient’s autonomy. The second aspect is that the disorder tends to worsen from generation to generation, with new and/or more severe symptoms possibly appearing in the same family. This latter aspect is particularly important from the perspective of providing adequate genetic counseling.

## Methods

### Data collection

Clinical, biochemical and genetic data were collected from medical records of six children from five different families diagnosed with Glut1-DS through exome sequencing analyses and followed at the Pediatric Neurology Unit at “Vittore Buzzi” Children’s Hospital, Milan, Italy. Genetic analyses were performed in the children’s relatives and 10 adults carrying pathogenic variants in *SLC2A1* were identified and included in the study.

Intelligence was assessed using Raven’s Standard Progressive Matrices (SPM) in adults and Raven’s Colored Progressive Matrices (CPM) in children [18]. SPM and CPM determine a subject’s nonverbal Intelligence Quotient. The final raw score is converted to a percentile falling into three different categories: low (< 25th percentile), normal (25th–75th percentile) and high (> 75th percentile).

An ad hoc interview was created to study life achievements, investigating educational aspects (difficulties experienced at school, support from special needs teachers, inclusion in rehabilitation programs, level of education attained), employment status (occasional, part-time or full-time job), autonomy (driving license), and social condition (marriage) (Additional file 1).

Quality of life was assessed using the World Health Organization Quality of Life questionnaire (WHOQOL-100, Italian version) [19]. The WHOQOL consists of 100 questions relating to six general domains (physical domain, psychological domain, level of independence, social relationships, environment, and spirituality). It provides six QoL domain profiles that are translated into a final score of 0 to 100, reflecting the importance attached by patients to the different aspects of their life. The results were used to establish the extent to which Glut1-DS affects these patients’ daily lives and routines. For the purpose of comparing QoL in the adult clinical population (9 patients) with a non-clinical population, the same questionnaire was administered to a sample of 18 sex- and age-matched healthy adults without any chronic disorder or neuropsychiatric disease, enrolled to obtain a case–control ratio of 1:2.

Tests and questionnaires were administered between April 2021 and May 2021, either face to face or, because of pandemic-related restrictions, remotely.

### Statistical analysis

The authors performed a descriptive analysis of all of the variables in the case–control dataset: continuous variables were summarized using median and quartiles (Q1–Q3) because of the higher robustness of these indices with respect to small sample size and possible outliers. Categorical variables were reported using absolute frequencies and percentages. Descriptive statistical indices were reported separately for adults and pediatric cases as well as for the case–control dataset.

Due to the small sample size and relatively large number of outcome variables (six QoL domain scores), the authors performed a principal component analysis (PCA) on the six domains of QoL and the results were used to choose an adequate synthesis of the relative scores.

The primary objective was to evaluate, in the adult subgroup, associations of the six QoL domain scores (quantitative variables ranging from 0 to 100) with the following variables: IQ (classified as: low-average-high), paroxysmal exertion-induced dyskinesia (PED; classified as: Yes/No), fatigue (classified as: Yes/No). The associations were evaluated using Kruskal–Wallis non-parametric ANOVA tests with the aforementioned synthetic variable as the response variable and IQ, PED and fatigue as grouping variables.

Correlations across the six domains of QoL were evaluated using PCA analysis results based on Pearson’s correlation coefficient.

The secondary objective was to evaluate putative differences in QoL scores between cases and controls. Data on QoL scores were available for nine of the 10 adult cases considered and for the 18 controls enrolled.

To this end, conditional logistic regression models were implemented using patient status (classified as: case/control) as the response variable and the single QoL domain scores as explanatory variables. Results were reported as estimated ORs with respective 95% confidence intervals and Wald test of association.

Finally, a PCA was performed in order, considering the six domains, to identify possible patterns of QoL in the two groups (Glut1-DS and healthy subjects) as well as to evaluate correlations between these features; in this context the variables (QoL domains) and the cases and controls were represented graphically using a biplot [20].

All hypothesis tests were two-tailed and the level of significance was set at α = 0.05.

All analyses were performed using the statistical software R (version 4.1.2) [21].

## Supplementary Information

**Additional file 1**. Interview. Description: Ad hoc interview used to study life achievements, investigating educational aspects, employment status, autonomy, and social condition.

**Additional file 2**. Variables factor map resulting from PCA performed on the adult subgroup dataset. Description: The 6 QoL domains are represented by arrows and they show a tendency toward the right side of the graph and dimension 1 of the PCA.

**Additional file 3**. Pearson’s correlation coefficients matrix relative to PCA performed on 9 adult patients. Description: Correlations across the 6 QoL domains are shown here. The Pearson’s correlation coefficients ranging from 0.38 up to 0.96.

**Additional file 4**. Boxplot of PED and QoL. Description: In order to evaluate the relationship between PED and QoL we performed a Kruskal-Wallis rank sum test; the result was not statistically significant (p =0.121) and the interpretation of this analysis has little meaning as only 1/9 patients reported no PED-related impairment.

**Additional file 5**. Relationship between Fatigue and QoL. Description: We performed a Kruskal-Wallis rank sum test, there are no statistically significant differences (p-value=0.439) nor does the graph suggest a substantial difference in the distribution between subjects with and without "fatigue" with respect to the QoL variable.

## Data Availability

Anonymized data supporting the conclusion of this study are available from the corresponding author (P.V.) on reasonable request. Not all data are publicly available because they contain information that could compromise patients´ privacy.

## Abbreviations

Glut1-DS: Glucose transporter type 1 deficiency syndrome
MDs: Movement disorders
PED: Paroxysmal exercise-induced dyskinesia
QoL: Quality of life
CSF: Cerebrospinal fluid
KD: Ketogenic diet
ADHD: Attention deficit hyperactivity disorder
ASM: Anti-seizure medication
SPM: Standard progressive matrices
CPM: Colored progressive matrices
WHOQOL: World Health Organization quality of life questionnaire
IQR: Interquartile range
PCA: Principal component analysis
QoL-S: 6-Items QoL domain
IQ: Intelligence quotient
OR: Odds ratio
