# Supplementary material for: Glut1 deficiency syndrome throughout life: clinical phenotypes, intelligence, life achievements and quality of life in familial cases
Source: Orphanet J Rare Dis. 2022 Sep 24;17:365. doi: 10.1186/s13023-022-02513-4 (PMC9509642; doi:10.1186/s13023-022-02513-4)
Supplement: Supplementary file 1 — Additional file 1. Interview. Description: Ad hoc interview used to study life achievements, investigating educational aspects, employment status, autonomy, and social condition. [file 13023_2022_2513_MOESM1_ESM.docx]

**Supplementary material 1: interview**

INTELLECTUAL DISABILITY:

-  Have you ever been administered a cognitive test?

-  Which qualification do you have (education)?

-  Qualification:

-  Did you have a special needs teacher?

-  Did you have any difficulties at school?

-  Do you have a driver’s license?

 Have you ever performed any types of rehabilitation programs (such as psychomotricity, speech therapy)?

-  If so, how long have you performed it?

SOCIAL LIFE:

-  Are you married?

-  Do you have any sons or daughters? How are they?

-  Do you have a job?

-  If so, what do you do? Is it part time or full time?

-  Are you in a specific protected category?

-  Are you followed by social services?
