# Supplementary figures and images for: Glut1 deficiency syndrome throughout life: clinical phenotypes, intelligence, life achievements and quality of life in familial cases
Source: Orphanet J Rare Dis. 2022 Sep 24;17:365. doi: 10.1186/s13023-022-02513-4 (PMC9509642; doi:10.1186/s13023-022-02513-4)

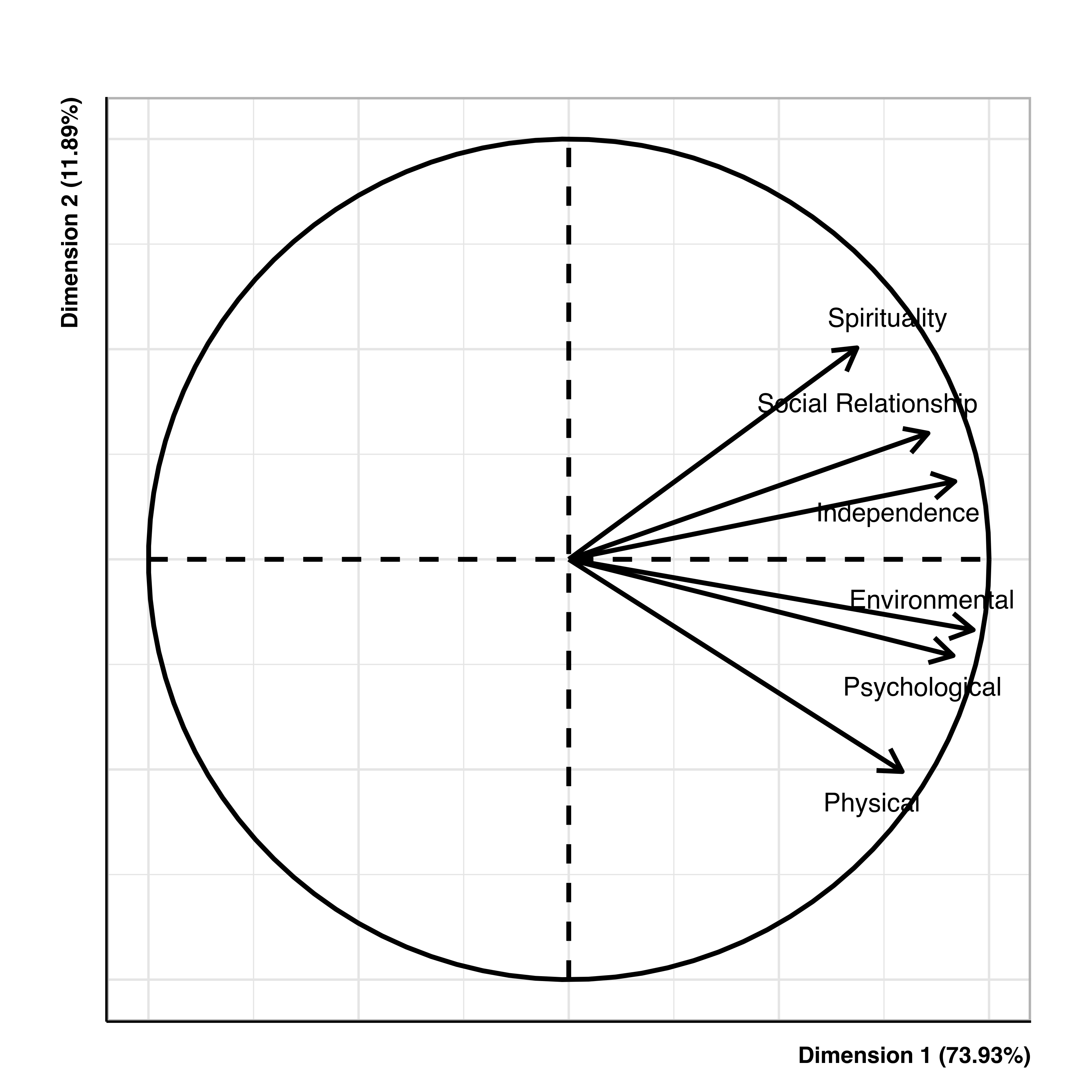

Supplement: Supplementary file 2 — Additional file 2. Variables factor map resulting from PCA performed on the adult subgroup dataset. Description: The 6 QoL domains are represented by arrows and they show a tendency toward the right side of the graph and dimension 1 of the PCA. [file 13023_2022_2513_MOESM2_ESM.tiff]

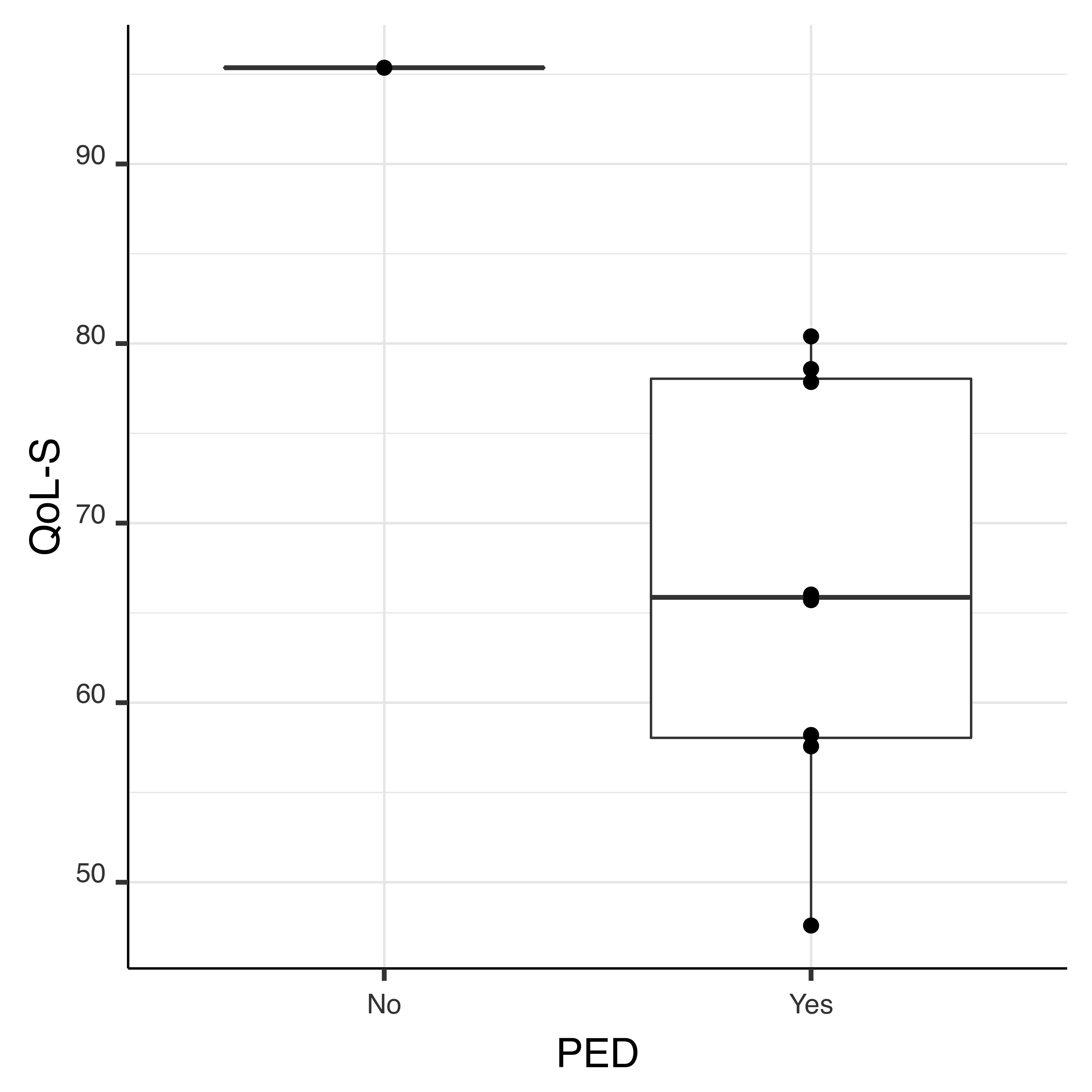

Supplement: Supplementary file 4 — Additional file 4. Boxplot of PED and QoL. Description: In order to evaluate the relationship between PED and QoL we performed a Kruskal-Wallis rank sum test; the result was not statistically significant (p =0.121) and the interpretation of this analysis has little meaning as only 1/9 patients reported no PED-related impairment. [file 13023_2022_2513_MOESM4_ESM.tiff]

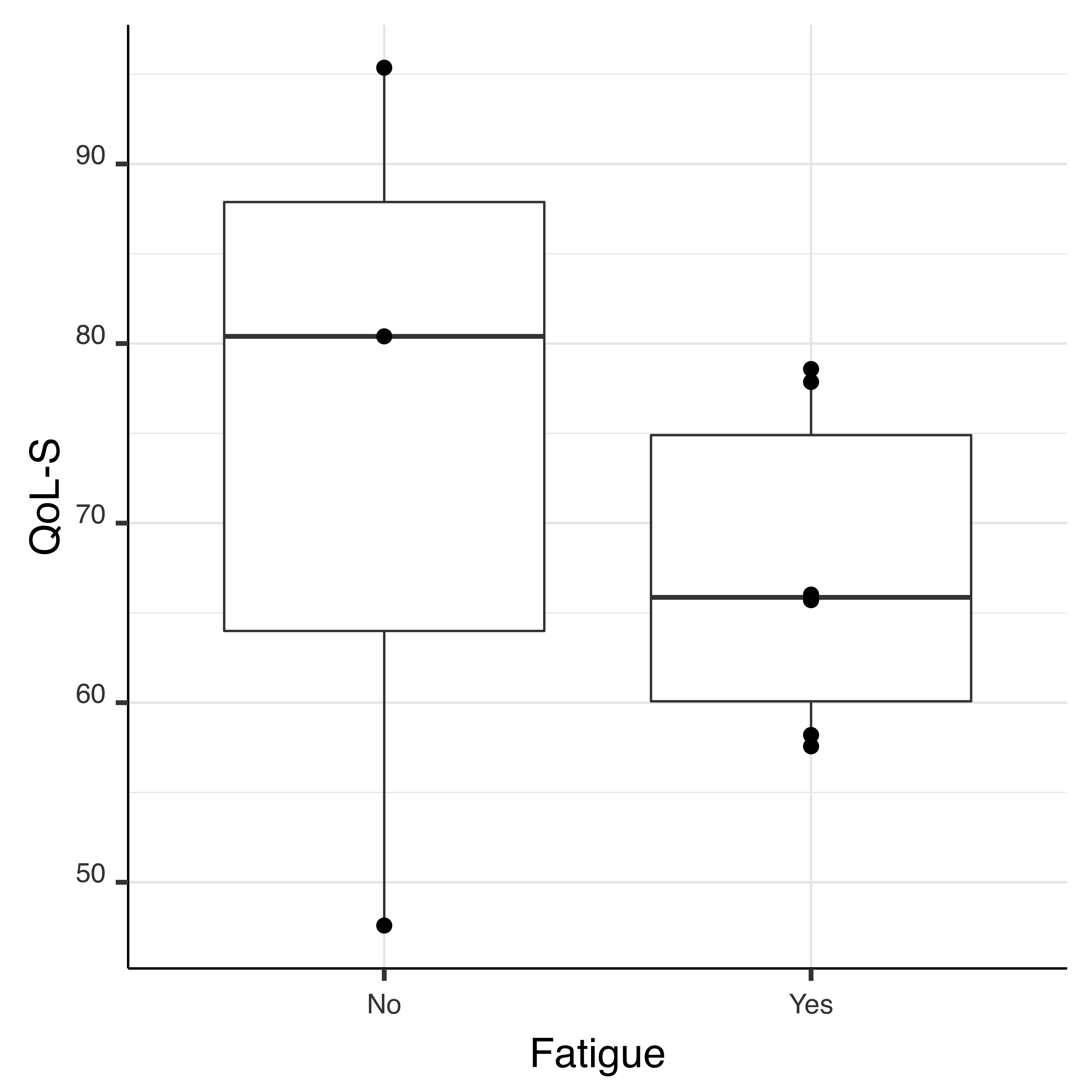

Supplement: Supplementary file 5 — Additional file 5. Relationship between Fatigue and QoL. Description: We performed a Kruskal-Wallis rank sum test, there are no statistically significant differences (p-value=0.439) nor does the graph suggest a substantial difference in the distribution between subjects with and without "fatigue" with respect to the QoL variable. [file 13023_2022_2513_MOESM5_ESM.tiff]
