# Supplementary material for: Glut1 deficiency syndrome throughout life: clinical phenotypes, intelligence, life achievements and quality of life in familial cases
Source: Orphanet J Rare Dis. 2022 Sep 24;17:365. doi: 10.1186/s13023-022-02513-4 (PMC9509642; doi:10.1186/s13023-022-02513-4)
Supplement: Supplementary file 3 — Additional file 3. Pearson’s correlation coefficients matrix relative to PCA performed on 9 adult patients. Description: Correlations across the 6 QoL domains are shown here. The Pearson’s correlation coefficients ranging from 0.38 up to 0.96. [file 13023_2022_2513_MOESM3_ESM.docx]

**Supplementary material 3. Pearson’s correlation coefficients matrix relative to PCA performed on 9 adult patients.**

|  | **Physical Domain** | **Psychological Domain** | **Independence Domain** | **Social Relationship Domain** | **Environmental Domain** | **Spirituality Domain** |
| --- | --- | --- | --- | --- | --- | --- |
| **Physical Domain** | 1.00 | 0.75 | 0.65 | 0.48 | 0.81 | 0.38 |
| **Psychological Domain** | 0.75 | 1.00 | 0.72 | 0.70 | 0.96 | 0.52 |
| **Independence Domain** | 0.65 | 0.72 | 1.00 | 0.93 | 0.81 | 0.59 |
| **Social Relationship Domain** | 0.48 | 0.70 | 0.93 | 1.00 | 0.74 | 0.53 |
| **Environmental Domain** | 0.81 | 0.96 | 0.81 | 0.74 | 1.00 | 0.61 |
| **Spirituality Domain** | 0.38 | 0.52 | 0.59 | 0.53 | 0.61 | 1.00 |

*Correlations across the 6 QoL domains are shown here. The Pearson’s correlation coefficients ranging from 0.38 up to 0.96.*
